# A toxic endophyte-infected grass helps reverse degradation and loss of biodiversity of over-grazed grasslands in northwest China

**DOI:** 10.1038/srep18527

**Published:** 2015-12-18

**Authors:** Xiang Yao, Michael J. Christensen, Gensheng Bao, Chunping Zhang, Xiuzhang Li, Chunjie Li, Zhibiao Nan

**Affiliations:** 1 The State Key Laboratory of Grassland Agro-ecosystems; College of Pastoral Agriculture Science and Technology, Lanzhou University, P. O. Box 61, Lanzhou 730020, Gansu, China

## Abstract

Overgrazing of China’s grasslands is increasingly causing biodiversity to decline. In degenerated grasslands of northwest China endophyte (*Epichloё gansuensis*) infected *Achnatherum inebrians* (drunken horse grass) is becoming widely distributed because of its toxicity to livestock. In this study, we investigated the ecological consequences of endophyte toxicity in this native grass, at three sites in northwest China, by comparing seed production of plant species and arthropod abundance in overgrazed grasslands with and without the presence of *A. inebrians.* Our findings demonstrate that the presence of endophyte infected *A. inebrians* reduces the loss of plant and arthropod biodiversity by providing a protected nursery free of animal grazing. Therefore, *A. inebrians*, typically regarded as an unwanted toxic invader by pastoralists, should be viewed as beneficial for grasslands as its presence maintains plant and arthropod biodiversity, and provides a foundation stone in the reconstruction and restoration of these grassland ecosystems.

Overgrazing of grasslands worldwide is causing flora and fauna biodiversity to decline^1^,^2^,^3^. China has the second largest area of pastoral land in the world, and these pastoral lands play a very important role in the global ecology^4^. Without exception, grasslands in northwest China face serious overgrazing from livestock, resulting in reduction of forage grasses, invasion of inedible plants, and appearance of bare ground^5^,^6^,^7^. In contrast, a native perennial grass species *Achnatherum inebrians*, known as “drunken horse grass”, is now thriving in these grasslands due to its toxicity to grazing animals such as sheep, goats, cattle and horses^8^,^9^. This toxicity is caused by a seed-transmitted symptomless fungal endophyte, *Epichloë gansuensis*^10^. *Epichloë* endophytes can be viewed as fungi that grow and function in host grasses as if they are a host tissue, with the growth being fully synchronized and symptomless within host plant tissue^11^.

*Achnatherum inebrians* is now widely found in grasslands of northwest China, including Qinghai province, Tibet province, Gansu province, Xinjiang province and parts of Inner Mongolia^9^. The grasses distribution has expanded as pastures have degenerated through overgrazing in past decades. For example, in Xinjiang, *A. inebrians* occupied 400 000 ha in 1987 and had expanded to 533 000 ha by 1992^9^. Due to its conspicuous appearance in pastures, that once had luxuriant forage grass, and its toxicity, *A. inebrians* has become a target for control by livestock owners^12^,^13^,^14^. However, the role of this toxic grass in these systems is poorly understood.

*Achnatherum inebrians* can reach a height of 60–150 cm and each compact inflorescence can produce about 700 small easily-shed seeds^9^. Based on field observations, *A. inebrians* forms discrete tussocks within pastures. Due to its morphology and toxicity, *A. inebrians* could provide a refuge from grazing animals, enabling the survival of palatable plant species. This could also have flow on effects to the arthropod members of the grasslands ecosystem.

Burrowing animals also are a feature of these grassland ecosystems. These include zokor (*Myospalax baileyi*) that produces molehills, and pika (*Ochotona curesezoniae*) that produce bare patches of ground within the grassland. Zokor are mainly located in eastern Asia^15^ while pika are widely distributed in Asia, Europe and North America^16^,^17^,^18^,^19^. These animals have been blamed for contributing to grassland degradation and are actively removed from grasslands^6^. *Achnatherum inebrians* appears to be a good colonizer of these bare patches and molehills whilst being rare in overgrazed areas that are still covered by vegetation.

The aim of this study was to determine if arthropod and plant biodiversity in areas with and without tussocks of *A. inebrians* differs and if bare ground produced by burrowing animals promotes *A. inebrians* establishment.

## Results

### Impact of *A. inebrians* on seed status of other plant species

Plant species present in grasslands of Xiahe, Guinan and Alxa generally, had a greater density of seeds per unit area when tussocks of *A. inebrians* (TA) were present, compared to areas without this toxic grass (NA). In Xiahe, an area dominated by *Elymus nutans*, there were 32 plant species that had mature seeds. Of these, 21 species had significantly higher inflorescence numbers or plant numbers in TA compared to NA, and 11 of these 21 species only produced seeds in TA. Whilst *A. inebrians* was generally correlated with greater seed density in TA, five species showed the opposite trend and six species showed no significant difference. Total quantity of seeds of all species and all forage grasses were significantly more abundant in TA than NA (Table 1).

In Guinan, a wet area dominated by the sedge *Kobresia capillifolia*, there were 12 species that had mature seeds, nine of which were only found in TA and had increased inflorescence or plant numbers. Interestingly, two species were unique to NA, but they were in relatively low abundance. Only one species had a significantly higher number of plants with seed in NA. Overall, the total quantity of seeds of all species and all forage grasses were significantly more abundant in TA than NA (Table 1).

In Alxa, an arid area dominated by the C_4_ grass *Pennisetum centrasiaticum*, five species, three of which only had seeds in TA, produced more abundant mature seeds, in TA compared to NA (Table 1).

### Impact of *A. inebrians* on arthropods

Most of the arthropod families identified from soil samples were found with significantly greater numbers in TA compared to NA. In Xiahe, of the 15 families of arthropods 12 were significantly greater in TA when compared with NA. Distribution of three arthropod families did not significantly differ between in the two treatments. Total quantity of individuals of all families was significantly more abundant in TA than NA (Table 2).

In Guinan, there were 11 families of arthropods that were unique to TA. No arthropods were identified in the NA pastures in this study. Nine of the 11 families had significantly greater number in TA than NA (Table 2).

In Alxa, there were 12 families of arthropods identified, seven of which were only found in TA, with 5 families being present in both treatments, only one of which was significantly increased in the TA sample. Total quantity of all arthropods was significantly greater in TA than NA (Table 2).

### Contribution of zokor and pika to the spread of *A. inebrians*

**Figure 1:**
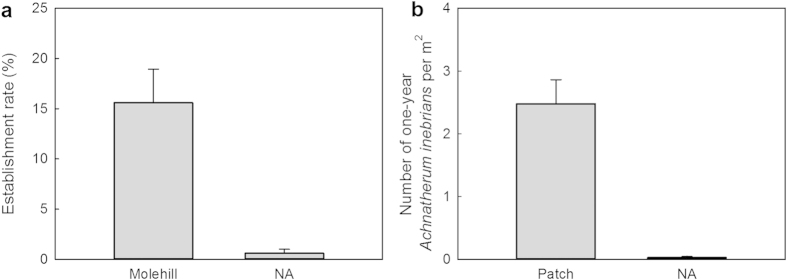
The spread of *Achnatherum inebrians*. (**a**) Comparison on the establishment rate of *A. inebrians* seeds in molehills and in overgrazed area without *A. inebrians* (NA) in Xiahe. (**b**) Comparison on number of one-year old *A. inebrians* plant in bare patches and overgrazed area without *A. inebrians* (NA) in Guinan. Mean values were presented in the text ± 1 SE.

The establishment rate from seeds of *A. inebrians* on molehills of zokor was significantly greater than in NA in Xiahe (Fig. 1a). The average density of one-year old *A. inebrians* plants in bare patches was significantly greater than in NA in Guinan (Fig. 1b).

## Discussion

*Achnatherum inebrians* is likely to play a positive role in the protection and regeneration of overgrazed grassland ecosystems in northwest China since it was shown in this study to be able to colonise bare ground. The growth habit of *A. inebrians* is such that large tussock plants develop interspaced with other palatable pasture species which can become established due to the production by *A. inebrians* of endophyte toxins that deter livestock grazing. This sheltered environment becomes a nursery area where the protected plants can flourish and again produce seed. Therefore, rather than being an undesirable invader^12^,^13^,^14^, this study has revealed that *A. inebrians* provides a means by which the seed resources of edible plant species can be saved in the face of over-grazing.

Without bare soil *A. inebrians* has a poor ability to establish from seed. The bare land in which *A. inebrians* can establish and which is now a widespread feature of degenerated grasslands, is frequently the result of the feeding activity of burrowing animals^18^. A common viewpoint is that the bare patches caused by pika and the molehills of bare earth caused by zokor are a major cause of grasslands degeneration^6^. Zokor and pika are widely distributed in grasslands and have a high density in the Qinghai-Tibetan Plateau (QTP)^20^,^21^. Bare patches caused by pika accounted for 62.6% of the total area of degraded grassland in Qinghai Province, while 25% of the total area was degraded in the QTP^18^. However, it is likely that continual overgrazing by livestock is primarily responsible for the conspicuous damage that these rodents are now causing. As suggested by Harris^22^, the presence of abundant rodents may be an indicator rather than a cause of grasslands degradation. Maybe overgrazing weakens the plant cover, even leading to the eradication of some plants and making the land more prone to bare patch formation. With a reduction in the concentration of suitable plants for food^23^, it seems that these small animals have increased their intensity of burrowing^24^ and also the area of burrowing activity in order to obtain sufficient food. These activities of the small animals facilitated the spread of *A. inebrians*, which can reach a height of 60–150 cm that is close to the height of livestock and produce about 700 small easily-shed seeds from each compact inflorescence^9^. The seed also has long awns that can adhere to livestock which may aid dispersal.

*Achnatherum inebrians* plants alone will not provide a stable ecosystem and this study has shown that within and between these large plants, members of the grasslands plant community also become established and have mature seeds. For a period of time after the onset of overgrazing there will remain a viable seed-bank in the soil and this may be the source of many of the new plants. However, continuous overgrazing will reduce the density and richness of forage seeds in soil^25^,^26^,^27^. Plants within and between the protective *A. inebrians* tussocks were shown in this study to produce more abundant seed compared with overgrazed areas. Some of the seeds may fall on the ground and so enrich the local seed-bank. In addition, some seed may move with the wind while other seed may be consumed by birds and thus get long distance dissemination^28^. These seeds will regenerate the broad-species seed-bank essential for maintaining a long-term stable ecosystem.

Arthropod diversity and abundance are also enhanced by the presence of *A. inebrians*. Part of the reason for the increased presence of arthropods is likely to be due to the open soil structure with high plant residues and organic matter content within *A. inebrians* protected pasture. In contrast, soil is generally very compacted in degraded pasture. In addition, the abundance of leaves will provide a food source for herbivorous arthropods^29^. Further, the high numbers of inflorescences provides pollen that is also a food source for some arthropods^30^ and so further increases arthropod biodiversity. The abundant arthropods and seeds will also become a food source of birds^28^,^31^.

The grasslands sites studied in the research ranged from a very dry area (Alxa) dominated by a C_4_ grass, to a wet area (Guinan) dominated by a sedge species. *Achnatherum inebrians* had become naturally established in this diverse range of habitats. This ability to colonise under such varied conditions makes this grass an effective agent for the regeneration of diverse degraded grasslands.

A further example of ecological impacts from another toxin-producing *Epichloë* species (*E. coenophialia*) that infects tall fescue (*Lolium arundinaceum*)^32^ has shown different effects on biodiversity. When endophyte-infected tall fescue was introduced to a pasture ecosystem by densely planting, the density and diversity of other plant species was reduced compared with tall fescue that is endophyte-free^33^. Endophyte-infected tall fescue also significantly reduced diversity of arthropods compared with endophyte-free plants^34^,^35^. However, one study suggested that there was no difference on effect on biodiversity between endophyte-infected and endophyte-free of tall fescue, but environmental condition played more important role^36^. Further research has also shown that compared with endophyte-free, endophyte-infected *Achnatherum robustum* and *Festuca arizonica* can increase biodiversity^37^,^38^,^39^, and endophyte-infected *L. multiflorum* strengthened interactions of resource-consumers^40^.

All of above studies compared different ecological roles between endophyte-infected and endophyte-free grass, but ignored the relationship between grass-endophyte symbiosis and livestock. A common practice to recover degenerated grassland is to use wire fences to prevent grazing livestock from exacerbating grassland degradation. However, wire fences also hindered the migration of wild animals^41^,^42^. In contrast, our research has demonstrated that *A. inebrians* can function as a natural fence which allows free movement of animals whilst naturally deterring grazing from damaging livestock. As such *A. inebrians* can be very helpful in the recovery of grassland.

In conclusion, *A. inebrians* can play a positive role in the stabilization and restoration of degenerated grasslands in northwest China. In addition, the effects of burrowing animals, implicated by many as a causal factor for grasslands degradation^6^, may in fact be beneficial in the restoration of pastures by providing suitable bare land for *A. inebrians* to establish.

## Methods

### Site descriptions

We conducted this study at three grasslands in northwest China that have been overgrazed by livestock in recent decades, and where *Achnatherum inebrians* plants have become increasingly more abundant. Xiahe county of Gansu province (35°07′ N, 102° 26′ E), Guinan county of Qinghai province (35° 28′ N, 101° 16′E) and Alxa Left Banner county, Inner Mongolia (38° 39′ N, 105° 46′E). In Xiahe, the dominant grass is *Elymus nutans*; Guinan, a wetland dominated by *Kobresia capillifolia*; Alxa, an arid grassland where *Pennisetum centrasiaticum*, a C_4_ grass, is the dominant species. Details of the three sites, including temperature and precipitation data, were presented in Supplementary Table S 1.

### Experimental designs

The entire study was performed in 2013, with the field assessments carried out between August 22^nd^ and September 21^st^. *Achnatherum inebrians* plants in all locations were assessed and determined to be endophyte infected using the method of Bacon *et al.*^32^.

### Impact of *A. inebrians* on seed status of other plant species

**Figure 2:**
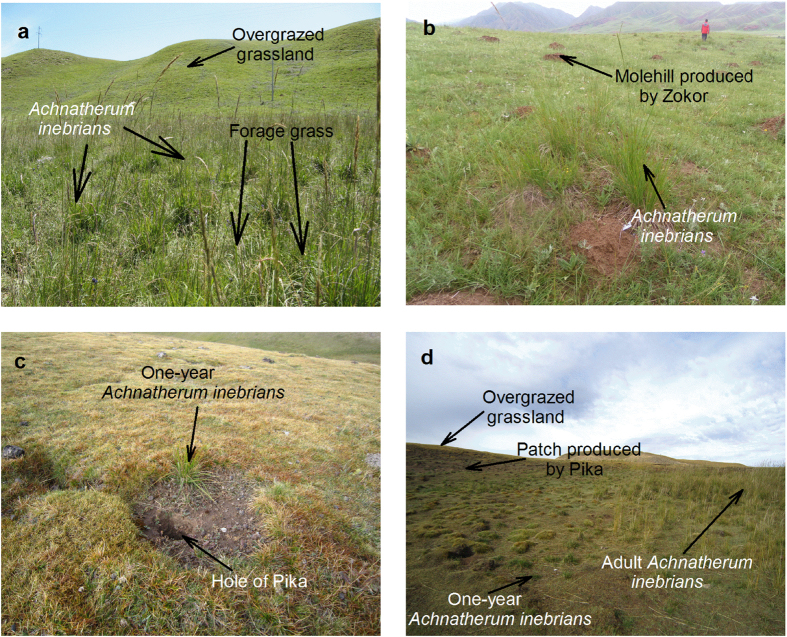
The protection on other plant species from *Achnatherum inebrians* and its spread. (**a**) Compared with those plant species that almost had no seeds and grew with low height in overgrazed grassland area, forage grasses and other plant species are abundant and have many inflorescences with mature seeds within tussocks of *A. inebrians* in Xiahe. (**b**) *Achnatherum inebrians* could easily establish in molehills that produced by zokor and widely distributed in overgrazed grassland in Xiahe. (**c**) Small *A. inebrians* plant established in a bare patch nearby a pika hole in overgrazed grassland in Guinan. (**d**) Many one-year old *A. inebrians* plants that had established in bare patches produced by pika in Guinan.

In order to determine the effects of *A. inebrians* on other plant species in the three sites, we employed two treatments in overgrazed grassland of each site: one area with tussocks of *A. inebrians* in the quadrats (5 m ×5 m), termed tussock area (hereafter TA) and the other one without *A. inebrians*, termed non-*A. inebrians* area (hereafter NA) that was still covered by short vegetation, and each treatment had four replications. The seed status of plants in each quadrat was recorded. We only investigated plants that had mature seeds. The inflorescence number for Poaceae and Cyperaceae species, because of relatively uniform inflorescences, and the plant number of plant species in other families with seeds were counted by visual observation in every quadrat *in situ*. Difference of vegetation between TA and NA can be seen in Fig. 2a.

### Impact of *A. inebrians* on arthropods

In each overgrazed grassland in Xiahe, Guinan and Alxa, 30 soil blocks (0.4 m in diameter and 0.2 m in depth, 15 each for the TA and NA quadrats) were randomly dug out and crumbled. All arthropods except for ants in one block were put in one collection bottle. The arthropod collections were brought to the laboratory and then the specimens were identified to the family level and counted.

### Contribution of zokor and pika to the spread of *A. inebrians*

A seed establishment experiment was conducted in grassland in Xiahe. Seeds of *A. inebrians* collected from Xiahe grassland in September 2012 were used in this study. Fifteen blocks were randomized in the overgrazed grassland, with each block having two small circular plots approximately 15 cm in diameter. One plot was on a zokor molehill and the other one was in adjacent overgrazed area (NA) that was still covered by short vegetation. These small plots were away from each other by at least 1 m. The sowing time was in April 2013, and in each plot 100 seeds were sown. The establishment rate of *A. inebrians* in each of the small plots was assessed in September 2013. Given the presence of *A. inebrians* in previously bare area produced by pikas, the ability of *A. inebrians* naturally colonising in these bare patches was investigated. This study was conducted in the grassland trial site of Guinan county. Only those *A. inebrians* plants that had established within the past year (less than one-year old plants), were investigated in this study to calculate the increase of plant number of *A. inebrians* in one year. Some areas covered by *A. inebrians* were randomly selected to compare the establishment ability of *A. inebrians* in bare patches and overgrazed grassland areas (NA) that were still covered by short vegetation. Those areas containing *A. inebrians* were not only close to bare patches but also close to NA areas. For each treatment, four quadrats (10 m ×10 m) were randomly selected in the overgrazed grassland. Calculation of the mean number of one-year old *A. inebrians* plants per m^2^ was carried out in every quadrat. The establishment ability of *A. inebrians* was compared by the mean number per m^2^ in the two treatments. The difference in establishment between bare land (molehill and patch) and NA can be seen in Fig. 2b–d.

### Data analysis

Data analysis was performed with SPSS 17.0 for Windows (USA). A nonparametric, Mann-Whitney U test was used to test: 1) the impact of *A. inebrians* on seed status of different plants species in two treatments; 2) the impact of *A. inebrians* on number of different arthropods in two treatments. Independent samples T-test was used to test: 3) the establishment rates of *A. inebrians* in molehill produced by zokor and NA; 4) the number of one-year old *A. inebrians* per m^2^ in bare patch produced by pika and NA.

## Additional Information

**How to cite this article**: Yao, X. *et al.* A toxic endophyte-infected grass helps reverse degradation and loss of biodiversity of over-grazed grasslands in northwest China. *Sci. Rep.* **5**, 18527; doi: 10.1038/srep18527 (2015).

## Supplementary Material

Supplementary Table S 1

## Figures and Tables

**Table 1:** Comparison of mature seed abundance of plant species in TA and NA.

Sites	Species	TA	NA	*P*-value
Xiahe	*Poa pratensis*^*①*^	206.75 ± 56.62^*^	12.00 ± 1.68	0.020
	*Elymus nutans*^*①*^	198.50 ± 48.16^*^	12.50 ± 6.84	0.020
	*Elymus sibiricus*^*①*^	143.00 ± 2.64^*^	0.00 ± 0.00	0.018
	*Leymus secalinus*^*①*^	77.75 ± 10.77^*^	0.25 ± 0.25	0.018
	*Stipa bungeana*^*①*^	36.25 ± 7.03^*^	9.25 ± 1.93	0.020
	*Stipa aliena*^*①*^	17.50 ± 3.23^*^	0.25 ± 0.25	0.018
	*Koeleria cristata*^*①*^	16.00 ± 3.94^*^	1.50 ± 0.96	0.020
	*Festuca sinensis*^*①*^	10.75 ± 2.06^*^	0.25 ± 0.25	0.018
	*Heteropappus altaicus*	362.25 ± 54.86^*^	97.25 ± 8.76	0.021
	*Melissitus ruthenica*	93.00 ± 9.17^*^	0.00 ± 0.00	0.047
	*Artemisia frigida*	50.33 ± 3.48^*^	0.00 ± 0.00	0.047
	*Artemisia scoparia*	49.00 ± 8.69^*^	19.25 ± 7.54	0.021
	*Limonium otolepis*	37.67 ± 6.36^*^	5.33 ± 4.37	0.013
	*Gueldenstaedtia verna*	24.67 ± 4.10^*^	0.00 ± 0.00	0.047
	*Taraxacum mongolicum*	21.00 ± 2.31^*^	5.67 ± 3.18	0.018
	*Dracocephalum heterophyllum*	18.50 ± 2.78^*^	2.00 ± 1.00	0.018
	*Chenopodium glaucum*	16.00 ± 3.87^*^	0.00 ± 0.00	0.014
	*Swertia mussotii*	7.75 ± 1.89^*^	0.00 ± 0.00	0.014
	*Geranium wilfordii*	6.75 ± 1.75^*^	0.00 ± 0.00	0.014
	*Elsholtzia densa*	5.50 ± 1.04	0.75 ± 0.48	0.457
	*Delphinium caeruleum*	5.25 ± 1.11^*^	0.50 ± 0.29	0.014
	*Potentilla anserina*	4.75 ± 1.03^*^	0.00 ± 0.00	0.013
	*Artemisia sieversiana*	4.25 ± 1.11	0.00 ± 0.00	0.064
	*Thalictrum alpinum*	3.00 ± 0.82	0.00 ± 0.00	0.130
	*Artemisia hedinii*	0.75 ± 0.75	0.50 ± 0.50	0.850
	*Clematis tangutica*	0.50 ± 0.50	0.00 ± 0.00	0.317
	*Leontopodium nanum*	0.00 ± 0.00	3.33 ± 0.33^*^	0.046
	*Thermopsis lanceolata*	0.00 ± 0.00	3.67 ± 0.67^*^	0.046
	*Anaphalis lactea*	0.00 ± 0.00	4.33 ± 0.88^*^	0.047
	*Euphorbia fischeriana*	0.00 ± 0.00	4.67 ± 0.88	0.317
	*Bupleurum smithii*	0.00 ± 0.00	5.00 ± 1.22^*^	0.013
	*Allium sikkimense*	0.00 ± 0.00	10.25 ± 1.80^*^	0.014
In total	Total seed abundance	1417.00 ± 103.69^***^	198.00 ± 15.16	*Pt* < 0.001
	Total seed abundance of forage plant	706.50 ± 108.85^***^	37.00 ± 5.78	*P*_tf_ < 0.001
Guinan	*Poa pratensis*^*①*^	756.25 ± 74.14^*^	0.00 ± 0.00	0.014
	*Leymus secalinus*^*①*^	63.00 ± 12.81^*^	0.00 ± 0.00	0.014
	*Elymus nutans*^*①*^	60.50 ± 6.17^*^	0.00 ± 0.00	0.014
	*Poa crymophila*^*①*^	37.25 ± 6.42^*^	0.00 ± 0.00	0.014
	*Koeleria cristata*^*①*^	17.67 ± 2.40^*^	0.00 ± 0.00	0.047
	*Stipa purpurea*^*①*^	4.50 ± 1.32^*^	0.00 ± 0.00	0.014
	*Stipa aliena*^*①*^	0.00 ± 0.00	4.25 ± 0.75^*^	0.013
	*Kobresia capillifolia*^*①*^	0.00 ± 0.00	16.00 ± 4.38^*^	0.014
	*Elsholtzia densa*	34.67 ± 7.31^*^	0.00 ± 0.00	0.047
	*Lepidium apetalum*	28.33 ± 4.37^*^	0.00 ± 0.00	0.047
	*Heteropappus altaicus*	18.25 ± 3.86^*^	0.00 ± 0.00	0.014
	*Astragalus polycladus*	4.25 ± 2.53	71.75 ± 6.64^*^	0.020
In total	Total seed abundance	1024.50 ± 84.63^***^	92.00 ± 7.26	*P*_t_ < 0.001
	Total seed abundance of forage plant	939.00 ± 81.02^***^	20.25 ± 3.97	*P*_tf_ < 0.001
Alxa	*Pennisetum centrasiaticum*^①^	39.75 ± 11.46^*^	0.50 ± 0.29	0.019
	*Setaria viridis*^*①*^	24.00 ± 4.92^*^	1.00 ± 0.71	0.020
	*Agropyron cristatum*^*①*^	8.00 ± 1.78^*^	0.00 ± 0.00	0.014
	*Commelina diffusa*	2.67 ± 0.33^*^	0.00 ± 0.00	0.046
	*Conyza canadensis*	3.00 ± 0.58^*^	0.00 ± 0.00	0.047
In total	Total seed abundance	77.50 ± 10.78^***^	1.50 ± 0.96	*P*_t_ < 0.001
	Total seed abundance of forage plant	71.75 ± 10.42^***^	1.50 ± 0.96	*P*_tf_ < 0.001

Notes: Inflorescence number of family Poaceae and Cyperaceae was counted, which were labeled “^①^”; plant species in other families were counted by plant number. *P*_t_ indicates difference of total seed abundance of all plant species in TA and NA in the three sites; *P*_tf_ indicates difference of total seed abundance of all edible forage plants in TA and NA in the three sites; “^*^” means *P* < 0.05; “^**^” means *P* < 0.01; “^***^” means *P* < 0.001; TA means areas with tussock of A. *inebrians*; NA means overgrazed areas without *A. inebrians*.

**Table 2:** Comparison of number of arthropods in TA and NA.

Sites	Family	TA	NA	*P*-value
Xiahe	Scarabaeidae(larvae)	10.53 ± 2.59	9.93 ± 1.95	0.380
	Tenebrionidae	4.60 ± 1.03^**^	0.40 ± 0.27	0.003
	Carabidae	3.47 ± 0.69^**^	0.10 ± 0.10	0.001
	Elateridae(larvae)	2.80 ± 0.66	0.70 ± 0.34	0.456
	Staphylinidae	1.80 ± 0.50^**^	0.00 ± 0.00	0.001
	Miridae	0.80 ± 0.21^**^	0.00 ± 0.00	0.007
	Curculionidae	0.670 ± 0.30^*^	0.00 ± 0.00	0.016
	Henicopidae	0.670 ± 0.21^**^	0.00 ± 0.00	0.007
	Clubionidae	0.60 ± 0.22^*^	0.00 ± 0.00	0.035
	Thomisidae	0.60 ± 0.22^*^	0.00 ± 0.00	0.016
	Coreidae	0.60 ± 0.22^*^	0.00 ± 0.00	0.016
	Lithobiidae	0.60 ± 0.22^*^	0.00 ± 0.00	0.016
	Labiduridae	0.53 ± 0.17^*^	0.00 ± 0.00	0.016
	Pentatomidae	0.47 ± 0.17^*^	0.00 ± 0.00	0.016
	Cerambycidae	0.20 ± 0.11	0.13 ± 0.09	0.550
In total	Total arthropod abundance	29.00 ± 4.26^***^	11.26 ± 1.82	*P*_t_ < 0.001
Guinan	Carabidae	2.53 ± 0.47^**^	0.00 ± 0.00	0.001
	Tenebrionidae	1.13 ± 0.46^*^	0.00 ± 0.00	0.017
	Scarabaeidae(larvae)	0.93 ± 0.23^**^	0.00 ± 0.00	0.003
	Chrysomelidae	0.73 ± 0.21^*^	0.00 ± 0.00	0.016
	Curculionidae	0.67 ± 0.22^*^	0.00 ± 0.00	0.017
	Staphylinidae	0.60 ± 0.22^*^	0.00 ± 0.00	0.016
	Nabidae	0.53 ± 0.09^*^	0.00 ± 0.00	0.017
	Lithobiidae	0.53 ± 0.22^*^	0.00 ± 0.00	0.035
	Elateridae(larvae)	0.53 ± 0.22^*^	0.00 ± 0.00	0.035
	Araneidae	0.20 ± 0.13	0.00 ± 0.00	0.150
	Philodromidae	0.13 ± 0.09	0.00 ± 0.00	0.317
In total	Total arthropod abundance	8.40 ± 2.53^***^	0.00 ± 0.00	*P*_t_ < 0.001
Alxa	Tenebrionidae	3.00 ± 0.79^**^	0.20 ± 0.20	0.004
	Porcellionidae	1.20 ± 0.63^*^	0.13 ± 0.09	0.025
	Dictynidae	1.20 ± 0.37^*^	0.20 ± 0.20	0.023
	Carabidae	1.13 ± 0.31^**^	0.00 ± 0.00	0.003
	Araneidae	0.93 ± 0.38^*^	0.00 ± 0.00	0.016
	Pyrrhocoridae	0.80 ± 0.36^*^	0.00 ± 0.00	0.035
	Staphylinidae	0.60 ± 0.16^**^	0.00 ± 0.00	0.007
	Coreidae	0.53 ± 0.17^*^	0.00 ± 0.00	0.016
	Lycosidae	0.27 ± 0.12	0.00 ± 0.00	0.073
	Elateridae	0.20 ± 0.11	0.00 ± 0.00	0.073
	Curculionidae	0.13 ± 0.09	0.07 ± 0.07	0.550
	Scarabaeidae(larvae)	0.07 ± 0.07	0.13 ± 0.09	0.550
In total	Total arthropod abundance	10.07 ± 2.06^***^	0.80 ± 0.41	*P*_t_ < 0.001

Notes: *P*_t_ indicates difference of total abundance of all arthropods families in TA and NA in the three sites; “^*^” means *P* < 0.05; “^**^” means *P* < 0.01; “^***^” means *P* < 0.001; TA means areas with tussock of A. *inebrians*; NA means overgrazed areas without *A. inebrians*.
