# Supplementary material for: A toxic endophyte-infected grass helps reverse degradation and loss of biodiversity of over-grazed grasslands in northwest China
Source: Sci Rep. 2015 Dec 18;5:18527. doi: 10.1038/srep18527 (PMC4683511; doi:10.1038/srep18527)
Supplement: Supplementary Table S 1 [file srep18527-s1.pdf]

Supplementary Information:

Title: A toxic endophyte-infected grass helps reverse degradation and loss of biodiversity of over-grazed grasslands in northwest China

Author list: Xiang Yao, Michael J. Christensen, Gensheng Bao, Chunping Zhang, Xiuzhang Li, Chunjie Li*, Zhibiao Nan

Supplementary Table S 1 Mean temperature (℃) and precipitation (mm) of sample areas in recent 5 years

	Mean temperature			Mean precipitation		
Year	Xiahe	Guinan	Alxa	Xiahe	Guinan	Alxa
2008	2.9	2.5	8.5	552	456	291
2009	3.6	3.2	9.6	413	506	135
2010	3.8	3.4	9.0	524	402	159
2011	3.2	2.6	8.6	583.	455	171
2012	3.0	2.3	8.5	646	591	233
